# The difference in CD4^+^ T cell immunity between high- and low-virulence Tembusu viruses is mainly related to residues 151 and 304 in the envelope protein

**DOI:** 10.3389/fimmu.2022.890263

**Published:** 2022-08-09

**Authors:** Runze Meng, Baolin Yang, Chonglun Feng, Jingjing Huang, Xiaoyan Wang, Dabing Zhang

**Affiliations:** Key Laboratory of Animal Epidemiology of the Ministry of Agriculture, College of Veterinary Medicine, China Agricultural University, Beijing, China

**Keywords:** Tembusu virus, virulence, cellular immune response, CD4^+^ T cell immunity, CD8^+^ T cell immunity

## Abstract

Tembusu virus (TMUV) can result in a severe disease affecting domestic ducks. The role of T cells in protection from TMUV infection and the molecular basis of T cell-mediated protection against TMUV remain largely uncharacterized. Here, we used the high-virulence TMUV strain Y and the low-virulence TMUV strain PS to investigate the protective role for TMUV-specific CD4^+^ and CD8^+^ T cells. When tested in a 5-day-old Pekin duck model, Y and PS induced comparable levels of neutralizing antibody, whereas Y elicited significantly stronger cellular immune response relative to PS. Using a duck adoptive transfer model, we showed that both CD4^+^ and CD8^+^ T cells provided significant protection from TMUV-related disease, with CD8^+^ T cell conferring more robust protection to recipient ducklings. For TMUV, CD4^+^ T cells mainly provided help for neutralizing antibody response, whereas CD8^+^ T cells mainly mediated viral clearance from infected tissues. The difference in T cell immunity between Y and PS was primarily attributed to CD4^+^ T cells; adoptive transfer of Y-specific CD4^+^ T cells resulted in significantly enhanced protective ability, neutralizing antibody response, and viral clearance from the brain relative to PS-specific CD4^+^ T cells. Further investigations with chimeric viruses, mutant viruses, and their parental viruses identified two mutations (T151A and R304M) in the envelope (E) protein that contributed significantly to TMUV-specific CD4^+^ T cell-mediated protective ability and neutralizing antibody response, with more beneficial effects being conferred by R304M. These data indicate T cell-mediated immunity is important for protection from disease, for viral clearance from tissues, and for the production of neutralizing antibodies, and that the difference in CD4^+^T cell immunity between high- and low-virulence TMUV strains is primarily related to residues 151 and 304 in the E protein.

## Introduction

Tembusu virus (TMUV) is an enveloped mosquito-borne flavivirus with a positive-sense, single-stranded RNA genome of approximately 11 kb. The RNA genome contains one large open reading frame (ORF), which is preceded by a 5’ untranslated region (UTR) and followed by a 3’ UTR. The ORF encodes ten polypeptides: three structural proteins [(capsid (C), precursor of membrane (prM), and envelope (E)] that form the viral particle and seven nonstructural proteins (NS1, NS2A, NS2B, NS3, NS4A, NS4B, and NS5) that are required for viral replication (1, 2).

TMUV can cause an acute infectious disease affecting ducks, which is of economic importance to all breeder duck farms, layer duck farms, and duck-growing farms (3–6). Affected ducks below 7 weeks of age develop signs of encephalitis consisting of ataxia, reluctance to walk, lameness, and paralysis (1, 4–7). Therefore, TMUV can be regarded as an encephalitic flavivirus. Experimental infections of Pekin ducklings (*Anas platyrhynchos domesticus*) less than 7 weeks of age have shown that the severity of TMUV-caused disease is associated with multiple factors, such as the age of birds, the route of application, the strain of virus, and the infectivity titer. In ducklings less than 9 days of age, TMUV generally causes fatal infection, with mortality ranging from 18% to 100% (1, 6, 8–11).

A better understanding of TMUV-induced adaptive immune response is crucial for the control of the TMUV-related disease. Through the use of plaque reduction neutralization test (PRNT), TMUV has been shown to elicit high, long-lasting neutralizing antibodies after natural infection and vaccination with attenuated vaccine (12). Previous works with E or prM/E-based subunit vaccine (13–16), DNA vaccine (17–19), and live vector vaccine (18–25) have demonstrated that the TMUV E and prM/E proteins induce neutralizing antibodies. The importance of E protein residue 408 in regulation of neutralizing antibodies has been highlighted by a recent investigation into attenuation-induced loss of immunogenicity (26). The C protein expressed by DNA vaccine was also shown to induce neutralizing antibodies (27).

Using duck models of TMUV infection, the TMUV-induced cellular immune response is beginning to be understood. TMUV induces significant up-regulation of IL-2 and IFN-γ at 7 days post infection (pi) (9, 28), and significant increases in numbers of CD4^+^ and CD8^+^ T cells at 5 days pi (29). The E, prM/E, and C proteins were shown to induce cellular immune response by measurement of the expression of cytokines (e.g., IL-2, IL-4, IL-6, IFN-γ, and TNF-α) or the change of CD4^+^ and CD8^+^ T cell numbers in ducks following immunization with subunit vaccine, DNA vaccine, and live vector vaccine (13, 14, 17, 19, 20, 22, 27).

To date, the role of T cells in protection from TMUV infection and the molecular basis of T cell-mediated protection against TMUV remain largely uncharacterized. Earlier works in our laboratory showed that the natural isolate Y and the plaque-purified strain PS, which exhibited distinct virulence in a 2-day-old Pekin duckling model, elicited comparable levels of neutralizing antibody (11). In this study, we describe the comparative studies on cellular immune responses to Y and PS. Our studies indicate that marked differences in inducing T cell responses exist between Y and PS. On this basis, the molecular determinants responsible for the differences in cellular immune responses observed between Y and PS are described.

## Materials and methods

### Ducks and cells

Newly hatched Pekin ducklings were derived from Peking duck breeding farm, Institute of Animal Sciences, Chinese Academy of Agricultural Sciences, Beijing, China. Their parents had never received TMUV vaccine. All ducklings were confirmed to be free of TMUV infection by testing serum samples using PRNT for antibodies to TMUV (12) and TMUV-specific real-time quantitative PCR (RT-qPCR) for viral RNA (30). In all cases, ducks in each group were reared separately in different isolators. BHK-21 cells were maintained at 37°C in Dulbecco’s modified Eagle’s medium (DMEM; Macgene, Beijing, China) supplemented with 10% fetal bovine serum (FBS; Macgene, Beijing, China), 100 U/ml penicillin, and 0.1 mg/ml streptomycin.

### Viruses

The BHK-21 cell-derived Y and PS strains of TMUV were isolated and propagated previously, which were shown to display high- and low-virulence for 2-day-old Pekin ducklings respectively (11). Parental backbone viruses rY and rPS were rescued from the full-length cDNAs of Y and PS respectively. Chimeric viruses rPS-YE with the Y entire E gene and rPS-YNS1-3’UTR with the Y NS1-3’UTR region as well as mutant viruses R38K, T151A, and R304M that have K38, A151, and M304 of the E protein of strain Y, respectively, were generated in the backbone of the rPS genome (**Supplementary Figure 1**). Information relating to the generation of these viruses were described previously (11). Stock viral titers were determined by plaque assay in BHK-21 cells as described previously (12) and are expressed as plaque forming unit (PFU) per ml.

To obtain working stocks, viruses were propagated in BHK-21 cells as described previously (12). For strains Y and PS, working stocks were prepared by four passages in BHK-21 cells. Briefly, BHK-21 cells were infected with viruses at a multiplicity of infection (MOI) of 0.1 PFU/cell at 37°C for 1 h. The cells were washed three times with phosphate-buffered saline (PBS), and maintenance medium consisting of DMEM supplemented with 2% FBS, 100 U/ml penicillin, and 0.1 mg/ml streptomycin was added. After incubation at 37°C for 60 h, the infected cell cultures were freeze-thawed three times and clarified by centrifugation and filtration.

### Duck experiments

The first experiment was conducted to investigate the pathogenicity of strain Y in Pekin ducklings with different ages, employing strain PS as a control virus. A total of 140 newly hatched ducklings were divided into seven groups (n=20). When the ducklings grew to 3, 5, and 7 days of age, they were inoculated with virus by intramuscular (im) route at a dose of 2×10^3^ PFU, respectively. The mock-infected control group received 0.2 ml of supernatant prepared from uninfected BHK-21 cells by im inoculation at 3 days of age. The ducklings were monitored for 15 days for mortality.

To systematically compare the virulence of Y and PS in 5-day-old Pekin ducklings, experimental infections were performed as described above. In each group (n=60), 20 ducklings were monitored for 15 days for signs of encephalitis and weight loss as well as mortality, and 40 ducklings were used for examination of gross lesions and sample collection. Signs of encephalitis were divided into 5-grade severities of no change, very mild, mild, moderate, and marked, giving scores 0, 1, 2, 3, and 4, respectively. The ducklings were weighed once every 2 days between 1 and 15 days pi. Three ducks were randomly selected from each group at 1, 3, 5, and 7 days pi, and sera and ethylenediamine tetraacetic acid (EDTA)-anticoagulated bloods were sampled for detection of viremia and counting of CD4^+^ and CD8^+^ T cells respectively. Subsequently, the selected ducks were euthanized, tissues (brain, spleen, and thymus) were collected for measurement of TMUV burden and expression of cytokines and T cell markers, and spleens were weighed. Tissues collected at 5 days pi were also used for examination of histopathological changes and viral antigens. At 9, 11, 13, and 15 days pi, serum samples of three ducks were collected from each group. These sera, together with those collected between 1 and 7 days pi, were used for detection of neutralizing antibodies. For isolation of Y- and PS-specific CD4^+^ and CD8^+^ T cells and naïve CD4^+^ and CD8^+^ T cells, the EDTA-anticoagulated blood samples were collected at 9 day pi from infected and uninfected ducklings.

To identify the protein associated with the difference in cellular immune response between Y and PS, chimeric viruses (rPS-YE and rPS-YNS1-3’UTR) and their parental viruses (rY and rPS) were used to infect 5-day-old ducklings (n=23) as described above. To identify the residues associated with the difference in cellular immune response between Y and PS, mutant viruses (R38K, T151A, and R304M) and their parental viruses (rY and rPS) were used to infect 5-day-old ducklings (n=23). In each case, a mock-infected control (n=23) was included, and tissues (brain and thymus) were sampled from three ducks in each group at 7 days pi for measurement of the expression of cytokines and T cell markers. For isolation of R38K-, T151A-, R304M-, rY-, and rPS-specific CD4^+^ and CD8^+^ T cells, the EDTA-anticoagulated blood samples were collected at 9 days pi from ducklings infected with mutant viruses and their parental viruses.

### Quantitation of viral loads in tissue and serum samples

Viruses in the tissue and serum samples were quantified using a RT-qPCR assay targeting the E gene. The tissue samples were processed as 20% homogenates in PBS, followed by centrifugation at 10,000 g for 10 min. RNA was extracted from 250 μl of each supernatant or serum using a TRIpure reagent (Aidlab, Beijing, China) and reverse transcribed using a M-MLV Reverse Transcriptase kit (Promega, Madison, USA), according to the manufacturer’s instructions. 5 μl of cDNA was mixed with 1 μl of each of forward and reverse primers [**Table 1**; (30)], 10 μl of 2×AceQ qPCR SYBR Green Master Mix (Vazyme, Nanjing, China), and 3 μl of ddH_2_O. The RT-qPCR was performed using the conditions reported previously (30).

**Table 1:** Primers used for measurement of TMUV RNA levels and expression of cellular immune-related genes in tissues of infected Pekin ducklings by RT-qPCR.

Target	Primer sequence (5′→3′)	Primer sequence (3′→5′)
TMUV E^a^	CGCTGAGATGGAGGATTATGG	ACTGATTGTTTGGTGGCGTG
**Duck IL-2**	TAGAAAACCTGGGAACAAGC	ATTTCTTCCTCCAAGGTGAC
**Duck IL-17**	TGCCTACGGGAAGGTGATAC	ATTGATGGGGATGGAGTTGA
**Duck IFN-α**	CCTCCCGCCAACGCCTTCTC	TGTGCGGCTTGCTGCGTGTC
**Duck IFN-β**	CGCAACCTTCACCTCAGCAT	TCTTCATCCGCCGTATTAGC
**Duck IFN-γ**	ACCTCGTGGAACTGTCAAAC	ACTGGCTCCTTTTCCTTTTG
**Duck CD4**	ATTTCAACGCCACAGCAGAT	CCCAGGAGGGTTAGCAGACA
**Duck CD8**	CCTGCTTGCTGCTTCTCATT	TTGGCACCTTGGGATTCATT
Duck GAPDH^b^	ATGAGAAGTATGACAAGTCC	ACTGTCTTCGTGTG TGGCT

a Reported previously (30).

b Reported previously (31).

### Histopathological examination

The tissue samples were fixed in 4% formalin at room temperature for 24h. Five-μm-thick paraffin-embedded sections were prepared by a standard protocol (32). After staining with hematoxylin eosin (H & E), the sections were checked for histopathological changes under an Olympus microscope (Olympus, Tokyo, Japan).

### Immunohistochemistry

Ten-μm-thick paraffin-embedded sections were prepared by a standard protocol (33). The sections were strained using mouse anti-TMUV E monoclonal antibody F3B4 (1:500 dilution in PBS) (34) and horseradish peroxidase (HRP)-conjugated goat anti-mouse IgG (1:500 dilution in PBS, Thermo Fisher Scientific, Shanghai, China). After counterstaining with hematoxylin, the sections were checked for viral antigen under an Olympus microscope (Olympus, Tokyo, Japan).

### Neutralization assay

Neutralizing antibodies in sera were detected using a previously reported PRNT (12). First, serial 10-fold dilutions of each heat-inactivated (56°C for 30 min) sera were mixed with an equal volume of virus, and incubated at 37°C for 1 h. Second, BHK-21 cells were inoculated with the virus-serum mixture and incubated at 37°C in a CO_2_ incubator 1 h for adsorption. Finally, the cell cultures were covered with overlay medium consisting of DMEM containing 1% low melting-point agarose (Macgene, Beijing, China) and 2% FBS, and the plaque assay was conducted. Antibody titer is expressed as 50% end point titer (neutralizing dose, ND_50_).

### Measurement of cytokine mRNA levels in tissues

RT-qPCR was applied to detect the expression of several cytokines, including interleukin 2 (IL-2), IL-17, gamma interferon (IFN-γ), and tumor necrosis factor-beta (TNF-β), and T cell markers, including CD4 and CD8. Sample processing, RNA extraction, and cDNA synthesis were the same as described above. 5 μl of cDNA was mixed with 1 μl of each of forward and reverse primers (**Table 1**) (31), 10 μl of 2×AceQ qPCR SYBR Green Master Mix (Vazyme, Nanjing, China), and 4.2 μl of ddH_2_O. Duck glyceraldehyde-3-phosphate-dehydrogenase (GAPDH) was used as an endogenous control. RT-qPCR was performed for GAPDH and each cytokine and T cell marker as follows: 95°C for 5 min, followed by 40 cycles at 95°C for 10 s and 60°C for 30 s, and extension at 72° for 60s. Relative expression was calculated for each cytokine using a 2^-△△ct^ method (35).

### Flow cytometry

Peripheral blood mononuclear cells (PBMCs) were isolated from the EDTA-anticoagulant blood samples (3ml/duck) using a duck lymphocyte isolation kit (P5720, Solarbio, Beijing, China), according to the manufacturer’s instructions. Following sucrose density gradient centrifugation, PBMCs that were located in a layer with relative density of 1.050–1.078 g/ml were harvested. The cells were washed three times and resuspended in 5 ml of PBS. CD4^+^ (or CD8^+^) T cells in PBMCs (3 ml/duck) were analyzed on a BD Arial Fusion flow cytometry (BD, Franklin, USA), using mouse anti-duck mAb MCA2478 (or mouse anti-duck CD8 mAb MCA2479) (Bio-Rad, Shanghai, China) and fluorescein isothiocyanate (FITC)-conjugated goat anti-mouse IgG (Thermo, Waltham, USA). Frequencies of CD4^+^ and CD8^+^ T lymphocytes in PBMCs were presented with FlowJo 7.6 software (BD, Franklin, USA). To prepare T cells for adoptive transfer, we conducted a fluorescence-activated cell sorting (FACS) on the BD Arial Fusion flow cytometry. CD4^+^ and CD8^+^ T cells were sorted from PBMCs.

### Adoptive T cell transfer

To compare the T cell-mediated immunity between Y and PS viruses, adoptive T cell transfer protocol was conducted using 3-day-old Pekin ducklings as recipients. Groups of 23 ducklings were inoculated by intravenous (iv) route with Y-specific CD4^+^, Y-specific CD8^+^ T cells, PS-specific CD4^+^, PS-specific CD8^+^ T cells, naïve CD4^+^ T cells, naïve CD8^+^ T cells (1×10^7^/duck), or PBS (2 ml/duck). 12 h later, the recipients were challenged by im route with TMUV Y at a dose of 2×10^3^ PFU. A mock-transferred, non-challenged group (control) was included, which was inoculated twice with PBS (2 ml/duck). The ducklings were monitored for signs of encephalitis, weight loss, and mortality. Serum samples of three ducklings were collected from each group between 1 and 15 days after challenge for measurement of neutralizing antibodies and viral RNA levels. At 7 days after challenge, three ducklings in each group were euthanized for measurement of spleen weight, and their tissues (brain, spleen, and thymus) were sampled for measurement of viral RNA levels and examination of histopathological changes.

To compare the T cell-mediated immunity between mutant viruses and their parental viruses, rY-, rPS-, T151A-, and R304M-specific CD4^+^ and CD8^+^ T cells were transferred into 3-day-old ducklings as described above. The ducklings in each group (n=23) were monitored for mortality. The serum samples of three ducklings were collected from each of groups receiving CD4^+^ T cells between 1 and 15 days after challenge for detection of neutralizing antibodies. The brain samples of three ducklings were collected from each of groups receiving CD4^+^ T cells at 7 days after challenge for measurement of viral RNA levels.

### Statistical analysis

All data were analyzed using GraphPad Prism software (version 5.0) (GraphPad Software Inc., San Diego, CA, United States). Survival curves were analyzed by the *Log-rank* test. Viral RNA levels, neutralizing antibody titers, and cytokine and T cell marker mRNA levels were analyzed by two-tailed Student *t* test. Body weight, spleen weight, and frequencies of CD4^+^ and CD8^+^ T cells in PBMCs were analyzed by two-way analysis of variance (ANOVA).

## Results

### TMUV Y exhibits different pathogenicity in pekin ducklings aged 3, 5, and 7 days

**Figure 1:**
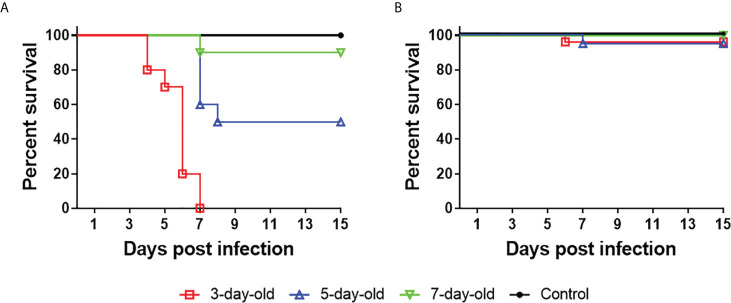
TMUV strain Y shows distinct pathogenicity in Pekin ducklings following infection at 3, 5, and 7 days of age. Groups of 20 ducklings were infected by an im route with virus at a dose of 2×10^3^ PFU, and monitored for mortality for 15 days. Shown are survival curves of ducklings infected with Y **(A)** and PS **(B)**. For ducklings infected with Y, significant differences in survival existed between 5-day-old and 7-day-old (*P*<0.01), 3-day-old and 5-day-old (*P*<0.001), 3-day-old and 7-day-old (*P*<0.0001) infected groups.

Earlier works in our laboratory have shown that following experimental infection of 2-day-old Pekin ducklings with 5 × 10^4^ PFU of Y or its rescued virus rY by intracerebral or subcutaneous routes, mortality as high as 90–100% occurred between 4 and 7 days pi (11), which is unfavorable for evaluation of immune responses against TMUV isolates. Thus, we assessed the pathogenicity of Y in 3- to 7-day-old Pekin ducklings by experimental infections using an im route and a lower infectivity titer (2×10^3^ PFU). All of the 20 3-day-old infected ducklings died between 4 and 7 days pi., 10 of the 20 5-day-old infected ducklings died between 7 and 15 days pi., and two of the 20 7-day-old infected ducklings died at 7 days pi **(** **Figure 1A** **)**. This suggests that Y exhibits moderate pathogenicity in 5-day-old Pekin duckling model relative to those observed in 3- and 7-day-old Pekin duckling models. PS retained low pathogenicity, similar to previously reported pathogenicity in 2-day-old Pekin duck model (11): 5% (1/20) mortality occurred following infection at 3 and 5 days of age, and no mortality was recorded in the case of 7-day-old infection **(** **Figure 1B** **)**. These data indicate that the marked differences in virulence between Y and PS can be retained in the 5-day-old Pekin duck model, with Y-induced pathogenic outcome being reduced as compared to that observed in 2- and 3-day-old Pekin duck models. Thus, use of the 5-day-old Pekin duckling model can ensure enough survivors at each time point after infection for testing of immune responses.

### TMUV Y and PS present different virulence in pekin ducklings aged 5 Days

**Figure 2:**
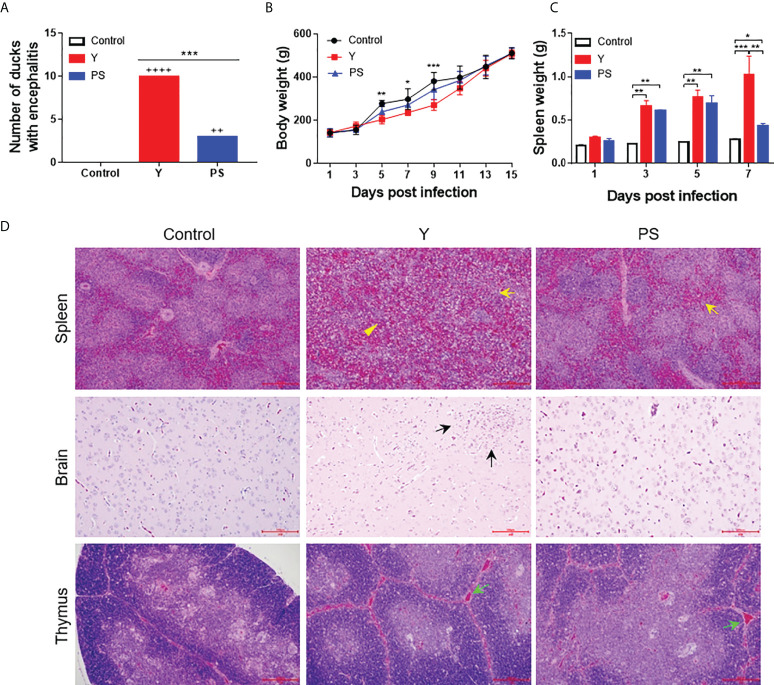
TMUV strain Y is more virulent in 5-day-old Pekin ducklings than strain PS. Groups of 60 ducklings were infected by an im route with virus at a dose of 2×10^3^ PFU or mock infected, and monitored for 15 days. **(A)** Number of infected ducklings displaying signs of encephalitis. ++, mild; ++++, marked. ***, *P*<0.001. **(B)** Body weight of infected ducklings. At each time point, data are presented as mean ± standard deviation (SD) of body weight from all surviving ducklings. Asterisks indicate significant differences between the group infected with Y and the controls (*, *P*<0.05; **, *P*<0.01; ***, *P*<0.001). **(C)** Spleen weight of infected ducklings. At each time point, data are presented as mean ± SD of spleen weight from three ducklings. *, *P*<0.05; **, *P*<0.01; ***, *P*<0.001. **(D)** Histopathological changes of infected ducklings. Shown necrotic vacuole (yellow arrows) and indistinct interface between red-pulp and white-pulp (yellow triangles) in spleen, inflammatory cell aggregation (black arrows) in brain, and increased interstitial hemorrhages of thymic corpuscles (green arrows) in thymus. Bar = 200 μm.

We further investigated the virulence of strains Y and PS in terms of clinical signs, mortality, and tissue injure using the 5-day-old Pekin duck model. Y caused severe signs of encephalitis within 5 to 9 days pi, including listlessness (10/20), tremor (6/20), and paralysis (5/20). In ducklings inoculated with PS, small portion (3/20) displayed signs of listlessness within 7 to 9 days pi (**Figure 2A**). Ducklings inoculated with Y and PS had 50% (10/20) and 5% (1/20) mortality respectively, like those observed above. Infection with Y affected weight gain between 9 and 13 days pi, with weight loss ranging from 18% to 31%, as compared to uninfected ducklings. Whereas no significant differences in body weight were detected between PS-infected ducklings and controls (**Figure 2B**). Infection with Y and PS both caused injury to spleen; however, we observed more severe gross lesions (more than 2-fold enlargement at 7 days pi; *P*<0.05; **Figure 2C**) and microscopic lesions (lymphocyte degeneration, necrosis, vacuolization, and depletion as well as indistinct interface between red and white pulp; **Figure 2D**, up panel) in spleens of ducklings inoculated with Y when compared to those observed in PS-infected ducklings. Microscopic lesions (perivascular lymphocyte infiltration) were observed in brains of ducklings inoculated with Y, whereas no microscopic lesions were detected in PS-infected ducklings (**Figure 2D**, middle panel). Similar microscopic lesions were seen in thymuses of ducklings infected with Y and PS (**Figure 2D**, bottom panel). These data confirm that infection with strain Y causes more severe disease relative to strain PS.

**Figure 3:**
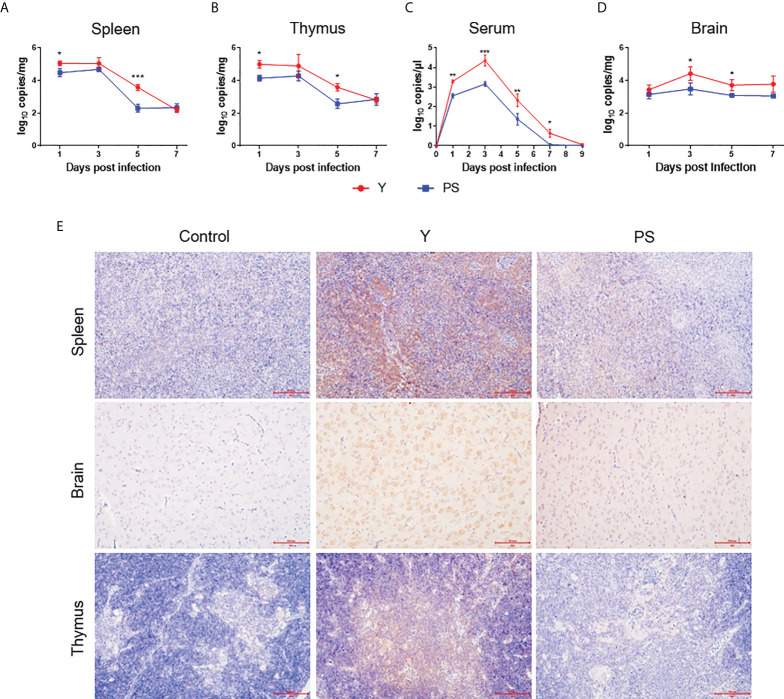
TMUV strain Y replicates in Pekin ducklings more efficiently than strain PS. Tissues were sampled from survivors in each group at different time points pi in experiments shown in **Figure 2**. Viral RNA levels in spleen **(A)**, thymus **(B)**, serum **(C)**, and brain **(D)** of three ducklings in each group were determined at different time points pi by the RT-qPCR assay. Data are presented as mean ± SD. *, *P*<0.05; **, *P*<0.01; ***, *P*<0.001. Tissues (brain, spleen, and thymus) collected at 5 days pi were subjected to immunohistochemical analysis **(E)**. Paraformaldehyde-fixed, paraffin-embedded tissues were immunolabeled with the TMUV E-specific mAb F3B4 and HRP-conjugated goat anti-mouse IgG.

To investigate the contribution of virus replication to virulence in 5-day-old Pekin ducklings, viral RNA levels were measured at different time points pi. During the whole observation period, viral RNA was detectable in all collected samples of infected ducklings. In general, levels of viral RNA in all samples of Y-infected ducklings were significantly higher than in those of PS-infected duckling. Y presented a similar replication kinetics in spleen and thymus, where similar levels of viral RNA were detected at a given time point pi; this was also the case for PS. However, viral RNA levels in Y- and PS-infected ducklings peaked at 1 and 3 days pi respectively. Moreover, Y produced approximately 4- and 16-fold-higher viral RNA levels in spleen and 7- and 10-fold-higher viral RNA levels in thymus at 1 and 5 days pi, respectively, relative to PS (**Figures 3A, B**). A similar viremia pattern was observed for Y and PS, both of which produced relatively high levels of viremia as early as 1 day pi and peak levels of viremia at 3 days pi; however, 3- to 24-fold-higher viral RNA levels were detected in Y-infected ducklings than in PS-infected ducklings between 1 and 7 days pi (**Figure 3C**). Viral RNA levels in brains of both Y- and PS-infected ducklings peaked at 3 days pi; however, Y produced 9- and 4-fold-higher viral RNA levels at 3 and 5 days pi, respectively, as compared to PS (**Figure 3D**). Immunohistochemical analysis of brain, spleen, and thymus revealed the presence of viral antigens in ducklings infected with both Y and PS. Whereas the immunolabeling was notably more intense in brain and more widespread in spleen in Y-infected ducklings than in PS-infected ducklings (**Figure 3E**). Collectively, our data indicate that the marked differences in virulence in 5-day-old Pekin duckling model between Y and PS is associated with their capacity to replicate in the periphery and the central nervous (CNS) and to produce and sustain the level of viremia.

### TMUV Y and PS induce similar levels of neutralizing antibody

**Figure 4:**
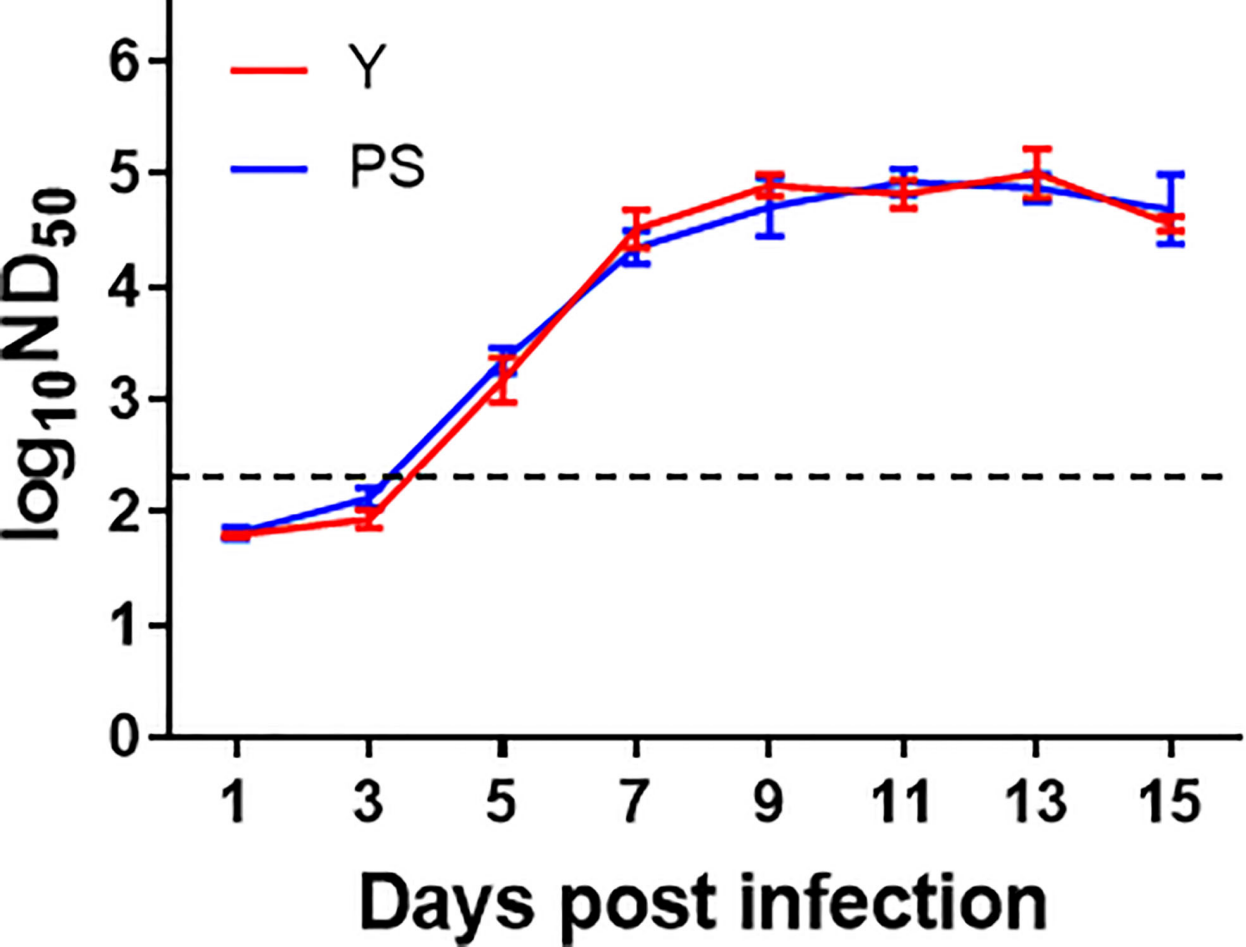
TMUV strains Y and PS elicit similar levels of neutralizing antibody in Pekin ducklings. Sera of survivors were collected from each group in experiments shown in FIGURE 2 were tested for neutralizing antibodies at different time points pi using PRNT. Dotted line indicates cut-off value defined recently for negative and positive sera (12). Data are presented as mean ± SD of the log_10_ ND_50_ from three ducklings.

Earlier works in our laboratory showed that strains Y and PS elicited comparable levels of neutralizing antibody in a 2-day-old Pekin duckling model (11). To provide further support to the neutralizing antibody responses induced by PS and Y, we repeated the neutralizing antibody analysis using the 5-day-old Pekin duckling model described above (**Figure 4**). A similar kinetics of neutralizing antibody response was observed for Y and PS, both of which elicited detectable neutralizing antibodies at 5 day pi that peaked at 9 days pi. No significant differences in neutralizing antibody between Y and PS were detected at any time point pi. Our data further confirm that TMUV strains Y and PS induce similar levels of neutralizing antibody response.

### TMUV Y induces higher magnitude of cellular immune responses than PS

**Figure 5:**
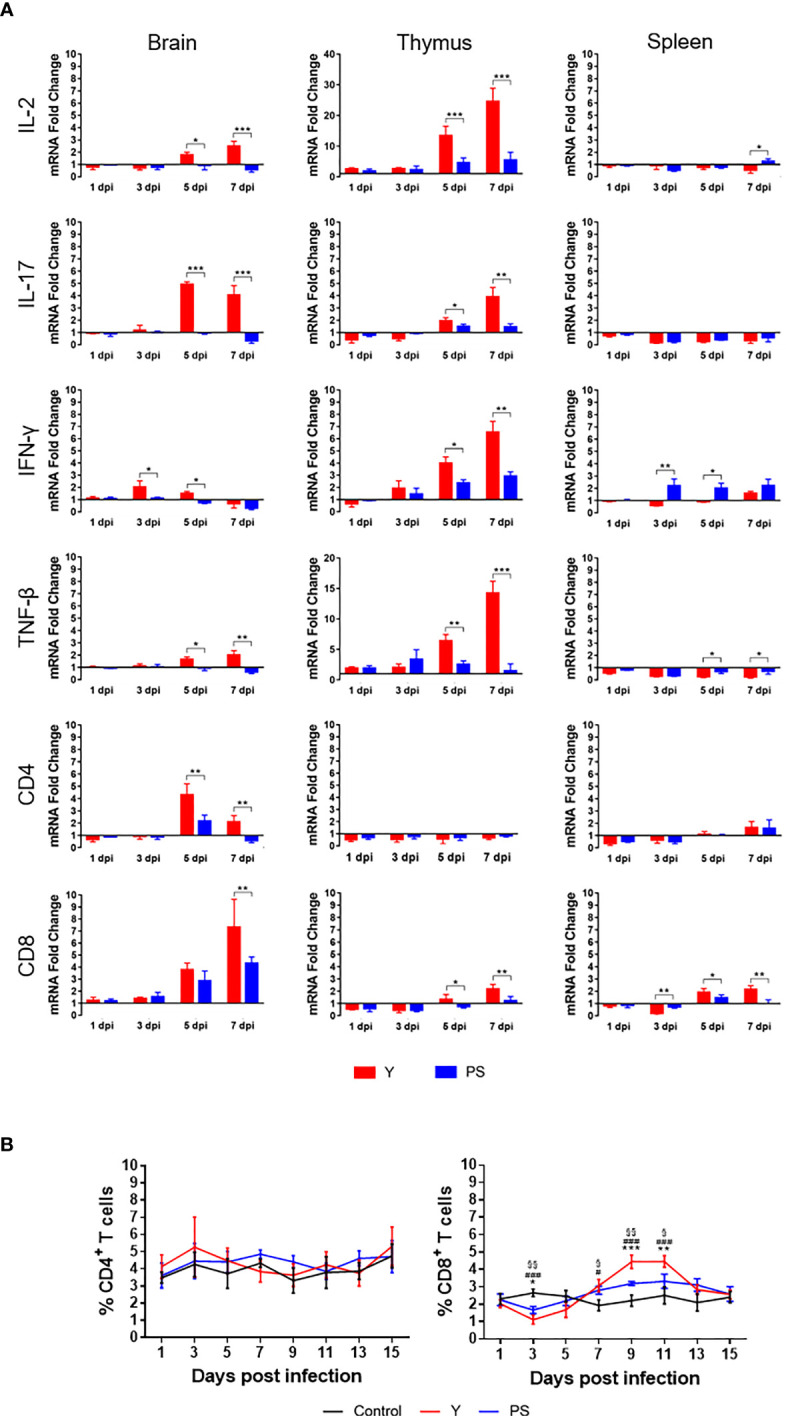
TMUV strain Y induces stronger cellular immune response in Pekin ducklings than strain PS. **(A)** Effect of infections with strains Y and PS on expression of cytokines (IL2, IL-17, IFN-γ, and TNF-β) and T cell markers (CD4 and CD8) in brain, thymus, and spleen of Pekin ducklings. Tissues of three survivors from each group were collected at different time points pi in experiments shown in FIGURE 2 and tested for relative expression of the cellular immune-related genes using RT-qPCR. Data are presented as means ± SD. *, *P*<0.05; **, *P*<0.01; ***, *P*<0.001. **(B)** Effect of infections with strains Y and PS on frequencies of CD4^+^ and CD8^+^ T cells in PBMCs of Pekin ducklings. Y- and PS-specific and naïve CD4^+^ and CD8^+^ T cells were isolated from PBMCs of three survivors from each group at different time points pi and counted. Data are presented as mean ± SD. Asterisks indicate significant differences between Y and PS viruses (*, *P*<0.05; **, *P*<0.01; ***, *P*<0.001). The # signs indicate significant differences between the groups infected with Y and the controls(#, *P*<0.05; ###, *P*<0.001). The § signs indicate significant differences between the groups infected with PS and the controls (§, *P*<0.05; §§, *P*<0.01; §§, *P*<0.001).

The marked differences in virulence and the similarity in neutralizing antibody responses between Y and PS suggest that there may be a link between the magnitude of cellular immune responses and TMUV virulence. To confirm the hypothesis, we measured the expression of cytokines (IL-2, IL-17, IFN-γ, and TNF-β) and T cell markers (CD4 and CD8) in brain, thymus, and spleen (**Figure 5A**) and the frequencies of CD4^+^ and CD8^+^ T cells in PBMCs (**Figure 5B**) at different time points pi. Infection with Y induced 2- to 13-fold increases in expression of cytokines and T cell markers tested in brain and thymus (except CD4 mRNA in thymus) at 5 and 7 days pi (3 and 5 days pi for IFN-γ response in brain; 7 days pi for CD8 expression in brain) (*P*<0.05), relative to infection with PS. These data indicate that strain Y induces stronger cellular immune response in brain and thymus than strain PS. Infection with Y induced 2- to 4-fold decreases in expression of IL-2 at 7 days pi, IFN-γ at 3 and 5 days pi, TNF-β at 5 and 7 days pi, and CD8 at 3 dpi (*P*<0.05) and 2- to 4-fold increases in CD8 expression at 5 and 7 dpi (*P*<0.05) in spleen, as compared to infection with PS, suggesting that strain Y exerts an inhibitory effect on expression of cytokines in spleen. Significant differences in frequency of CD8^+^ T cells between Y- and PS-infected ducklings were observed. Infection with Y resulted in more significant decrease in frequency of CD8^+^ T cells at 3 days pi (0.5-fold; *P*<0.05), and more significant increase in frequency of CD8^+^ T cells at 9 and 11 days pi (1-fold and 0.5-fold, respectively; *P*<0.05), relative to those observed for PS. These data reveal a negative correlation between TMUV virulence and frequency of CD8^+^ T cells at the peak of viremia and a positive correlation between TMUV virulence and frequency of CD8^+^ T cells after virus clearance from the circulation. Altogether, these data suggest that strain Y induces higher magnitude of cellular immune responses than PS, which can be reflected by measurement of expression of cytokines and T cell markers in brain and thymus of infected ducklings.

### Transfer of Y-specific T cells provide more significant protection relative to PS

**Figure 6:**
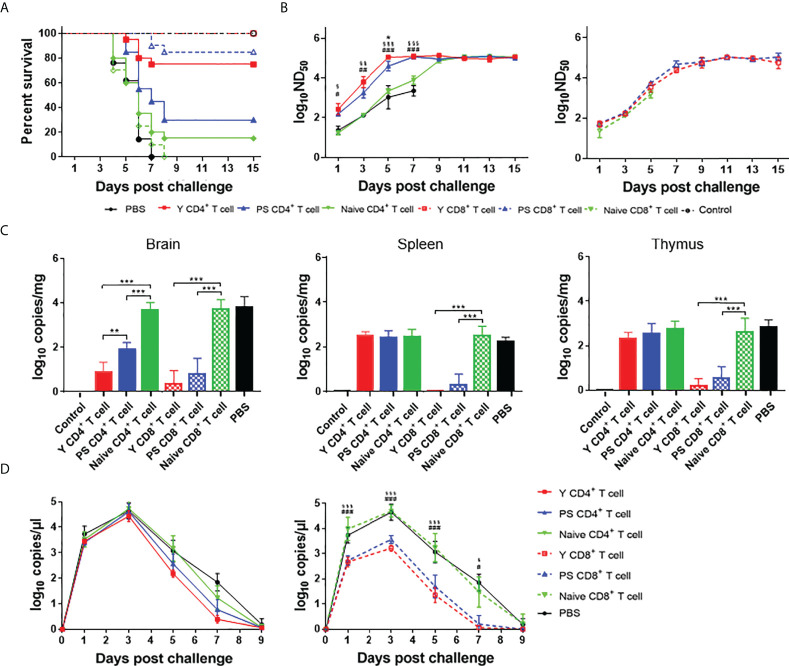
CD4^+^ and CD8^+^ T cells participate in protective response to TMUV. Groups of 23 3-day-old recipient ducklings were inoculated by iv route with Y-specific CD4^+^ T cells (Y CD4^+^ T cell), PS-specific CD4^+^ T cells (PS CD4^+^ T cell), Y-specific CD8^+^ T cells (Y CD8^+^ T cell), PS-specific CD8^+^ T cells (PS CD8^+^ T cell)), naïve CD4^+^ T cells (Naive CD4^+^ T cell), naïve CD8+ T cells (Naive CD8^+^ T cell) (10^7^/duck), or PBS. The recipient ducklings were challenged by im route with 2×10^3^ PFU of TMUV Y 12 h later, and monitored for 15 days. **(A)** Survival curves of recipient ducklings. Significant difference in survival was detected between groups received Y- and PS-specific CD4^+^ T cells (*P*<0.01); PS-specific and naïve CD4^+^ T cells (*P*<0.05); and Y-specific and naïve CD4^+^ T cells, Y-specific and naïve CD8^+^ T cells, and PS-specific and naïve CD8^+^T cells (*P*<0.0001). **(B)** Effect of transferred CD4^+^ (left) and CD8^+^ (right) T cells on the production of neutralizing antibodies in recipient ducklings. Neutralizing antibodies in sera were determined using PRNT. Data are presented as mean ± SD of the log_10_ ND_50_ from three ducklings. Asterisks indicate significant differences between groups received Y- and PS-specific T cells (*, *P*<0.05). The # signs indicate significant differences between Y-specific T cells adoptive groups and the control (#, *P*<0.05; ##, *P*<0.01; ###, *P*<0.001). The § signs indicate significant differences between PS-specific T cell adoptive group and the control (§, *P*<0.05; §§, *P*<0.01; §§, *P*<0.001). **(C)** Effect of transferred CD4^+^ and CD8^+^ T cells on viral RNA levels in tissues (spleen, thymus, and brain) of recipient ducklings at 7 days after challenge. Data are presented as mean ± SD of the log_10_ RNA copies per mg of tissue from three ducklings. **, *P*<0.01; ***, *P*<0.001. **(D)** Effect of transferred CD4^+^ and CD8^+^ T cells on viremia of recipient ducklings between 1 and 9 days after challenge. Data are presented as mean ± SD of the log_10_ RNA copies per μl for sera from three ducklings. The # signs indicate significant differences between Y-specific T cells adoptive group and the control (#, *P*<0.05; ###, *P*<0.001). The § signs indicate significant differences between PS-specific T cells adoptive group and the control (§, *P*<0.05; §§§, *P*<0.001).

To compare the protection conferred by Y- and PS-specific T cells, we performed adoptive cell transfer using 3-day-old Pekin ducklings as recipients. Contrasting with the high mortalities of ducklings received PBS (100%), naïve CD4^+^ T cells (90%), and naïve CD8^+^ T cells (100%), transfer of Y- and PS-specific CD8^+^ T cells and Y-specific CD4^+^ T cells provided significant protection (100%, 85%, and 75%, respectively) against lethal infection. Whereas PS-specific CD4^+^ T cells conferred only 30% protection to recipient ducklings (**Figure 6A**). Transfer of Y- and PS-specific CD4^+^ and CD8^+^ T cells was also effective in preventing signs of encephalitis, weight loss, and tissue injury upon TMUV Y infection, with Y-specific T cells being more effective than PS-specific T cells (**Supplementary Figures 2, 3**). Together, these data indicate that Y-specific T cells induce stronger protection against TMUV-related disease than PS-specific T cells and that for TMUV, CD8^+^ T cells induce stronger protection against TMUV-related disease than CD4^+^ T cells.

To investigate the contribution of Y- and PS-specific T cells in production of neutralizing antibodies, we determined neutralizing antibody titers between 1 and 15 days after challenge with strain Y. Significantly higher levels of neutralizing antibody were detected in ducklings received TMUV-specific CD4^+^ T cells between 1 and 7 days after challenge, as compared to those derived from ducklings received PBS and naïve CD4^+^ T cells. The neutralizing antibodies in ducklings received Y- and PS-specific CD4^+^ T cells peaked at 5 and 7 days after challenge, respectively. By comparison, the neutralizing antibodies in ducklings received naïve CD4^+^ T cells peaked at 11 days after challenge. In addition, significantly higher levels of neutralizing antibody were detected in ducklings received Y-specific CD4^+^ T cells between 3 and 5 days after challenge than in ducklings received PS-specific CD4^+^ T cells (**Figure 6B**, left panel). These data indicate that TMUV-specific CD4^+^ T cells provide help for neutralizing antibody response, whereas Y-specific CD4^+^ T cells contribute to a more beneficial effect on production of neutralizing antibodies than PS-specific CD4^+^ T cells. All ducklings received naïve CD8^+^ T cells died before 7 days after challenge. There was no significant difference in neutralizing antibody response between groups received TMUV-specific CD8^+^ T cells and naïve CD8^+^ T cells within 1 to 5 days after challenge, and between groups received Y- and PS-specific CD8^+^ T cells within 1 to 15 days after challenge (**Figure 6B**, right panel). These data indicate that Y- and PS-specific CD8^+^ T cells make no contribution to neutralizing antibody response.

To compare the effect of Y- and PS-specific T cells on viral load, we measured viral RNA levels in tissues (brain, spleen, and thymus) at 7 days after challenge and in serum samples between 1 and 9 days after challenge (**Figures 6C, D**). Adoptive transfer of Y- and PS-specific CD4^+^ T cells reduced viral RNA levels in brain (353-fold and 59-fold, respectively; *P*<0.001), and had no significant effect on viral RNA levels in spleen, thymus, and serum. By comparison, transferred Y- and PS-specific CD8^+^ T cells reduced viral RNA levels in brain (1549-fold and 590-fold, respectively; *P*<0.001), spleen (355-fold and 252-fold, respectively; *P*<0.001), and thymus (443-fold and 157-fold, respectively; *P*<0.001) and in serum (32- to 103-fold and 28- to 38-fold, respectively; *P*<0.001) between 1 and 5 days after challenge, and cleared virus from the blood at 7 days after challenge. There was no significant difference in viral RNA levels in brain, spleen, thymus, and serum between groups received Y- and PS-specific CD8^+^ T cells. Overall, our data suggest that virus-specific CD8^+^ T cells play an important role in clearing infection from tissues and preventing virus persistence, with Y- and PS-specific CD8^+^ T cells being similar in this function.

### The E protein plays a major role in determining the differences in cellular immune response between Y and PS

**Figure 7:**
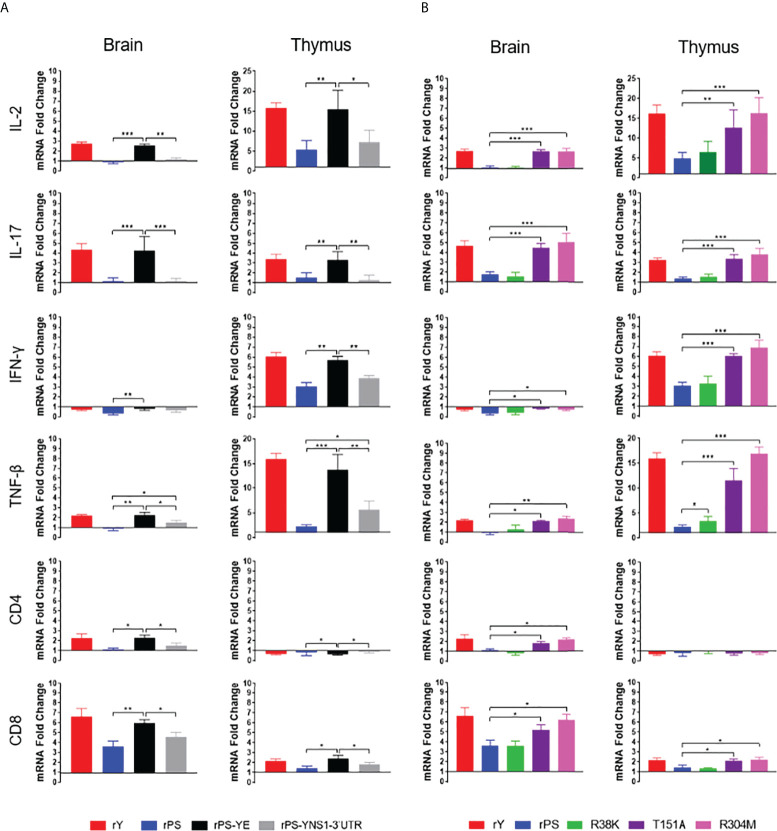
E protein residues 151 and 304 are responsible for the differences in cellular immune response between Y- and PS-infected Pekin ducklings. Groups of 23 5-day-old ducklings were inoculated with virus at a dose of 2×10^3^ PFU of virus or mock infected. **(A)** Expression of cellular immune-related genes in ducklings infected with chimeric (rPS-YE and rPS-YNS1-3’UTR) and control (rPS and rY) viruses. **(B)** Expression of cellular immune-related genes in ducklings infected with mutant (R38K, T151A, and M304R) and control (rPS and rY) viruses. The expression of the cellular immune-related genes in tissues (brain and thymus) of three individuals in each group was determined at 7 days pi using RT-qPCR. Data are represented as mean ± SD. *, *P*<0.05; **, *P*<0.01; ***, *P*<0.001.

To determine which protein is responsible for the differences in cellular immune response between Y and PS, 5-day-old Pekin ducks were infected with chimeric viruses rPS-YE and rPS-YNS1-3’UTR and control viruses rY and rPS. We measured the expression of IL-2, IL-17, IFN-γ, TNF-β, CD4, and CD8 mRNAs in brain and thymus at 7 days pi (**Figure 7A**). rY and rPS behaved like Y and PS (**Figure 5A**) respectively. Infection with rPS-YE significantly increased the levels of IL-2, IL-17, TNF-β, and CD8 in brain and thymus, CD4 in brain, and IFN- γ in thymus, and significantly reduced the levels of IFN-γ in brain and CD4 in thymus, as compared to those observed for the parental backbone virus rPS. As a result, rPS-YE infection induced mRNAs of these cytokines and T cell makers to similar levels as measured for rY. In general, infection with rPS-YNS1-3’UTR had little or no contribution to the expression of cytokines (except TNF-β) and T cell markers in brain and thymus (*P*<0.05). These data indicate that the E protein plays a major role in determining the differences in cellular immune response between Y and PS.

### The T151A and R304M mutations in the E protein contribute to significant enhanced cellular immune response to TMUV

We then identify E protein residues responsible for the differences in cellular immune response between Y and PS. 5-day-old Pekin ducklings were infected with the R38K, T151A, and R304M mutant viruses and the rY and rPS control viruses, and the levels of IL-2, IL-17, IFN-γ, TNF-β, CD4, and CD8 mRNAs in brain and thymus were measured at 7 days after infection (**Figure 7B**). In this experiment, rY and rPS also behaved like Y and PS (**Figure 5A**) respectively. Infections with both T151A and R304M significantly increased the levels of IL-2, IL-17, TNF-β, and CD8 in brain and thymus, IFN-γ in thymus, and CD4 in brain, as compared to infection with the rPS parental backbone virus (*P*<0.05). Thus, both T151A and R304M induced mRNAs of these cytokines and T cell makers to similar levels as measured for rY. Infection with R38K had little or no effect on expression of the tested cytokines (except TNF-β in thymus) and T cell markers in brain and thymus (*P*<0.05). Our data indicate that mutations T151A and R304M in the E protein contribute to the differences in cellular immune response between Y and PS.

### Mutations of E protein residues 151 and 304 affect CD4^+^ T cell-mediated immunity

Our studies indicate that the marked differences in CD4^+^ T cell-induced protection and neutralizing antibody response and some differences in CD8^+^ T cell-induced protection exist between Y- and PS-infected ducklings, which might be attributed to E protein residues 151 and 304. To confirm the hypothesis, CD4^+^ and CD8^+^ T cells from ducklings infected with mutant viruses T151A and R304M were used in the adoptive transfer experiments.

**Figure 8:**
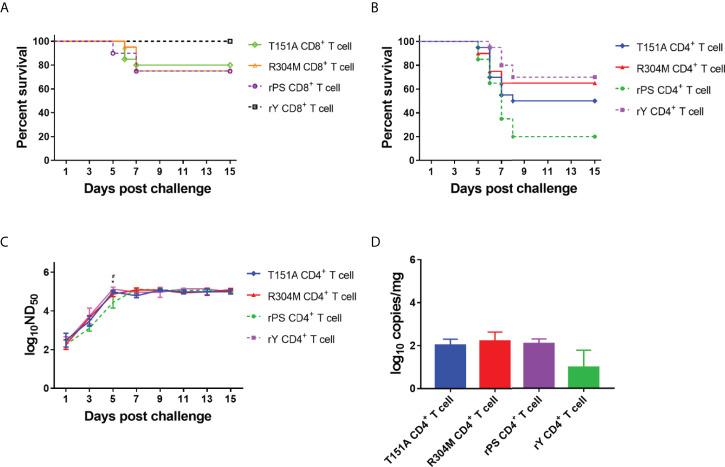
E protein residues 151 and 304 contribute to the differences in T cell-mediated immunity between Y- and PS-infected Pekin ducklings. Groups of 23 3-day-old recipient ducklings were inoculated by iv route with rY-specific CD4^+^ T cells (rY CD4^+^ T cell), rPS-specific CD4^+^ T cells (rPS CD4^+^ T cell), T151A CD4^+^ T cells (T151A CD4^+^ T cell), R304M CD4^+^ T cells (R304M CD4^+^ T cell), rY-specific CD8^+^ T cells (rY CD8^+^ T cell), rPS-specific CD8^+^ T cells (rPS CD8^+^ T cell)), T151A CD8^+^ T cells (Naive CD8^+^ T cell), R304M CD8^+^ T cells (R304M CD8^+^ T cell) (10^7^/duck). The recipient ducklings were challenged by im route with 2×10^3^ PFU of TMUV Y 12 h later, and monitored for 15 days. **(A)** Effect of transferred T151A- and R304M-specific CD8^+^ T cells on survival of recipient ducklings. **(B)** Effect of transferred T151A- and R304M-specific CD4^+^ T cells on survival of recipient ducklings. Significant difference in survival was detected between groups received T151A- and rPS-specific CD4^+^ T cells (*P*<0.05) as well as R304M- and rPS-specific CD4^+^ T cells (*P*<0.01) groups. **(C)** Effect of transferred T151A- and R304M-specific CD4^+^ T cells on neutralizing antibody response in recipient ducklings. Neutralizing antibodies in sera were determined using PRNT. Data are presented as mean ± SD of the log_10_ ND_50_ from three ducklings. Asterisks indicate significant differences between groups received T151A- and rPS-specific T CD4^+^ cells (*, *P*<0.05). The # signs indicate significant differences between R304M- and rPS-specific CD4^+^ducklings (#, *P*<0.05). **(D)** Measurement of TMUV burden in ducklings received CD4^+^ T cells. Viral RNA levels in brain of three ducklings in each group were determined at 7 days after challenge by the RT-qPCR assay. Data are presented as mean ± SD.

We evaluated adoptive transfer of CD8^+^ T cells from ducklings infected with mutant viruses T151A and R304M for its effect on protection of recipient ducklings from lethal challenge (**Figure 8A**). Survivals in groups received T151A-and R304M-specific CD8^+^T cells (80% and 75%, respectively) were comparable to those in group received rPS-specific CD8^+^ T cells (75%), but lower than those in group received rY-specific CD8^+^T cells (100%). The result indicates that the R304M and T151A mutations have little or no contribution to CD8^+^ T cell-mediated protection.

We assessed adoptive transfer of CD4^+^ T cells from ducklings infected with mutant viruses T151A and R304M for its effects on protective capacity, neutralizing antibody response, and TMUV burden in brain (**Figures 8B**–**D**). Varying degrees of increases in survival occurred in group received T151A- and R304M-specific CD4^+^T cells (50% and 65%, respectively), as compared to that in group received rPS-specific CD4^+^T cells (20%). The data indicate that both T151A and R304M mutations contribute to markedly enhanced CD4^+^ T cell-induced protective efficacy, with the R304M mutation contributing more to protection against lethality. Neutralizing antibodies in ducklings received rPS-specific CD4^+^T cells peaked at 7 days after challenge. By comparison, neutralizing antibodies in ducklings received T151A- and R304M-specific CD4^+^T cells peaked 2 days earlier, like that observed for rY-specific CD4^+^T cells. The levels of neutralizing antibody detected in ducklings received T151A-, R304M-, and rY-specific CD4^+^T cells between 1 and 5 days after challenge were similar, all of which were higher than those detected in ducklings received rPS-specific CD4^+^T cells, with significant higher antibody titers occurring at 5 days after challenge. These data indicate that both R304M and T151A mutations contribute to significantly increased CD4^+^ T cell-mediated neutralizing antibody response. The levels of viral RNA detected in brain of ducklings received T151A- and R304M-specific CD4^+^T cells at 7 days after challenge were similar to those derived from ducklings received rPS-specific CD4^+^T cells, indicating that both R304M and T151A mutations have no contribution to CD4^+^ T cell-mediated viral clearance from the brain.

## Discussion

The primary goal of the present study was to investigate the protective role for TMUV-specific CD4^+^ and CD8^+^ T cells by using the high-virulence TMUV strain Y and the low-virulence TMUV strain PS. Because strain Y caused very high mortality in recently reported 2-day-old Pekin duckling model (11), we first evaluated the pathogenicity of strain Y in 3, 5, and 7-day-old Pekin ducklings. We showed that strain Y presented distinct pathogenicity in 3-, 5-, and 7-day-old Pekin ducklings, indicating that the pathogenic outcome is age dependent even in the age range of 3 to 7 days. Strain Y presented a lower virulence phenotype in 5-day-old Pekin ducklings and a similarly high virulence phenotype in 3-day-old Pekin ducklings when compared with that observed in 2-day-old Pekin ducklings (11). Thus, the 5-day-old-Pekin duck model is a useful tool for the assessment of immune responses to TMUV strains with different virulence phenotypes, whereas the 3-day-old Pekin duckling model can be used as recipients in the adoptive transfer experiments.

The present observation confirmed recent findings in which TMUV strains Y and PS were shown to elicit comparable levels of neutralizing antibody in 2-day-old Pekin duck model (11). This indicates that the neutralizing antibody response fails to correlate with the difference in virulence between Y and PS. Considering that PS elicits significantly higher levels of neutralizing antibody than those observed for sufficiently attenuated PS180 strain derived from 180 passages of PS in BHK-21 cells (26), we can conclude that no correlation exists between the levels of neutralizing antibodies and virulence above a baseline level of low virulence represented by PS. In the investigation of Y- and PS-induced cellular immune responses, we observed more marked IL-2, IL-17, IFN-γ, and TNF-β responses and higher up-regulation of CD4 and CD8 genes at 5 and 7 days pi as well as more markedly increased frequencies of CD8^+^ T cells between 7 and 11 days pi in Y-infected ducklings than in PS-infected ducklings. These findings indicate that strain Y elicits stronger cellular immune response than PS, suggesting a positive correlation between the magnitude of TMUV-specific T cell immune response (especially CD8^+^ T cell response) and the virulence of TMUV.

We observed that the viral RNA levels in brain, thymus, spleen, and blood in both Y- and PS-infected ducklings tended to decline from 3 to 7 days pi, indicating that virus-induced immune responses have exerted their functions in inhibiting virus replication, limiting virus dissemination, clearing infection from tissues, and preventing viral persistence. The levels of viral RNA in Y-infected duckling were reduced to those in PS-infected ducklings at 7 days pi, suggesting a role of the excess of Y-specific T cell immunity over PS-specific T cell immunity in controlling TMUV burden. It is likely that more rapid clearance of virus from the blood of PS-infected ducklings than from Y-infected ducklings might be attributed to the role of glycosaminoglycan-binding motif at residue 304 in the E protein of PS. TMUV burden in brain of both Y- and PS-infected ducklings were reduced more slowly as compared to those in the extraneural tissues, which might be associated with immune responses in the CNS involve recruitment of peripheral immune cells to the CNS (36–38).

Using adoptive T cell transfer, we demonstrated that Y-specific CD4^+^ T cells and Y- and PS-specific CD8^+^ T cells provided significant protection against a lethal infection with TMUV Y, indicating a crucial role of T cells in the protective immune response to TMUV. CD8^+^ T cells were shown to confer more robust protection to recipient ducklings than CD4^+^ T cells, suggesting that for TMUV, similarly to dengue virus (DENV) (39, 40), CD8^+^ T cells are more relevant to the control of TMUV-related disease. It is interesting to observe a more marked difference in protection between Y- and PS-specific CD4^+^ T cells in comparison to that between Y- and PS-specific CD8^+^ T cells. This may indicate that the difference in T cell-mediated protective immune response between Y and PS is primarily attributed to CD4^+^ T cells.

The measurement of neutralizing antibodies in sera and viral RNA levels in tissues revealed that for TMUV, CD4^+^ T cells primarily made contribution to the production of neutralizing antibodies, and CD8^+^ T cells primarily mediated viral clearance for brain, thymus, spleen, and blood, similar to other flaviviruses (e.g., DENV, West Nile virus, Japanese encephalitis virus, and yellow fever virus) (39, 41–45). Nevertheless, TMUV-specific CD4^+^ T cells also mediated viral clearance from the brain, similar to DENV-specific CD4^+^ T cells (44). Whereas Y- and PS-specific CD4^+^ T cells were distinct from each other in terms of contribution to neutralizing antibody response and viral clearance from the brain. It is likely that the difference in T cell-mediated protective immune response between Y and PS can be attributed to the different contribution of Y- and PS-specific CD4^+^ T cell to the production of neutralizing antibodies and the clearance of virus from the brain.

From studies on expression of cytokines and T cell markers in ducklings infected with chimeric and mutant viruses we conclude that E protein residues 151 and 304 are the key determinants of the magnitude of TMUV-specific T cell immune response. Based on adoptive transfer of mutant virus-specific T cells, this work has also shown that E protein residues 151 and 304 are the key determinants of protection against lethal infection and neutralizing antibody production mediated by TMUV-specific CD4^+^ T cells. We observed that the contribution of both T151A- and R304M-specific CD4^+^ T cells to protection and antibody production failed to achieve the levels conferred by rY-specific CD4^+^ T cells, suggesting that a combination of the T151A and R304M mutations might synergistically enhance virus-specific CD4^+^ T cell immunity.

Previous studies with other flaviviruses (e.g., DENV and JEV) have shown that CD4^+^ T cells mainly target the E, C, and NS1 proteins, whereas CD8^+^ T cells preferentially recognize the NS3, NS4B, and NS5 proteins (46–52). We speculate that E protein residues 151 and 304 are likely to be located within one of epitopes recognized by TMUV-specific CD4^+^ T cells, that the difference in viral clearance from the brain between Y- and PS-specific CD4^+^ T cells might be associated with one or more of the three residues in the NS1 protein that differ between Y and PS (11), and that the difference in protection between Y- and PS-specific CD8^+^ T cells could be related to one or more of the 10 residues in NS2A, NS3, NS4B, and NS5 proteins that differ between Y and PS (11). Further studies are needed to confirm the hypothesis.

Taken together, our studies demonstrate a critical role of CD4^+^ and CD8^+^ T cells in the protective immune response and the control of TMUV infection. A positive correlation exists between the virulence of TMUV and T cell immunity, including CD8^+^ T cell-mediated protection and CD4^+^ T cell-mediated protection, neutralizing antibody response, and viral clearance from the brain. We have also demonstrated that the difference in CD4^+^ T cell-mediated immunity is mainly related to residues 151 and 304 in the E protein. Our studies contribute to the better understanding of the role of T cell immunity in the protective immune response and the molecular basis of TMUV-induced CD4^+^ T cell immunity.

## Data availability statement

The original contributions presented in the study are included in the article/**Supplementary Material**. Further inquiries can be directed to the corresponding author.

## Ethics statement

The animal study was reviewed and approved by Animal Welfare and Ethics Committee of China Agricultural University.

## Author contributions

RM conducted experiments, analysis, and writing of the original manuscript draft. BY, CF, and JH were involved in sample collection, sample processing, analysis, and data collection. XW was involved in the analysis and interpretation of data. DZ performed analysis, writing, and reviewing. All authors contributed to the article and approved the submitted version.

## Funding

This project was supported by grants from China Agriculture Research System of MOF and MARA for DZ and the National Key Research and Development Program of China (2016YFD0500107) for DZ.

## Acknowledgments

We thank Lixin Yang (China Agricultural University) for advice and suggestions in duck experiments; Duo Peng, Qiong Li, Jiaying Wang, and Zixin Feng (China Agricultural University) for their support in sample collection.

## Conflict of interest

The authors declare that the research was conducted in the absence of any commercial or financial relationships that could be construed as a potential conflict of interest.

## Publisher’s note

All claims expressed in this article are solely those of the authors and do not necessarily represent those of their affiliated organizations, or those of the publisher, the editors and the reviewers. Any product that may be evaluated in this article, or claim that may be made by its manufacturer, is not guaranteed or endorsed by the publisher.
