# Supplementary material for: The difference in CD4+ T cell immunity between high- and low-virulence Tembusu viruses is mainly related to residues 151 and 304 in the envelope protein
Source: Front Immunol. 2022 Aug 9;13:890263. doi: 10.3389/fimmu.2022.890263 (PMC9395619; doi:10.3389/fimmu.2022.890263)
Supplement: Supplementary file 1 [file DataSheet_1.docx]

***Supplementary Material***

## Supplementary Figures


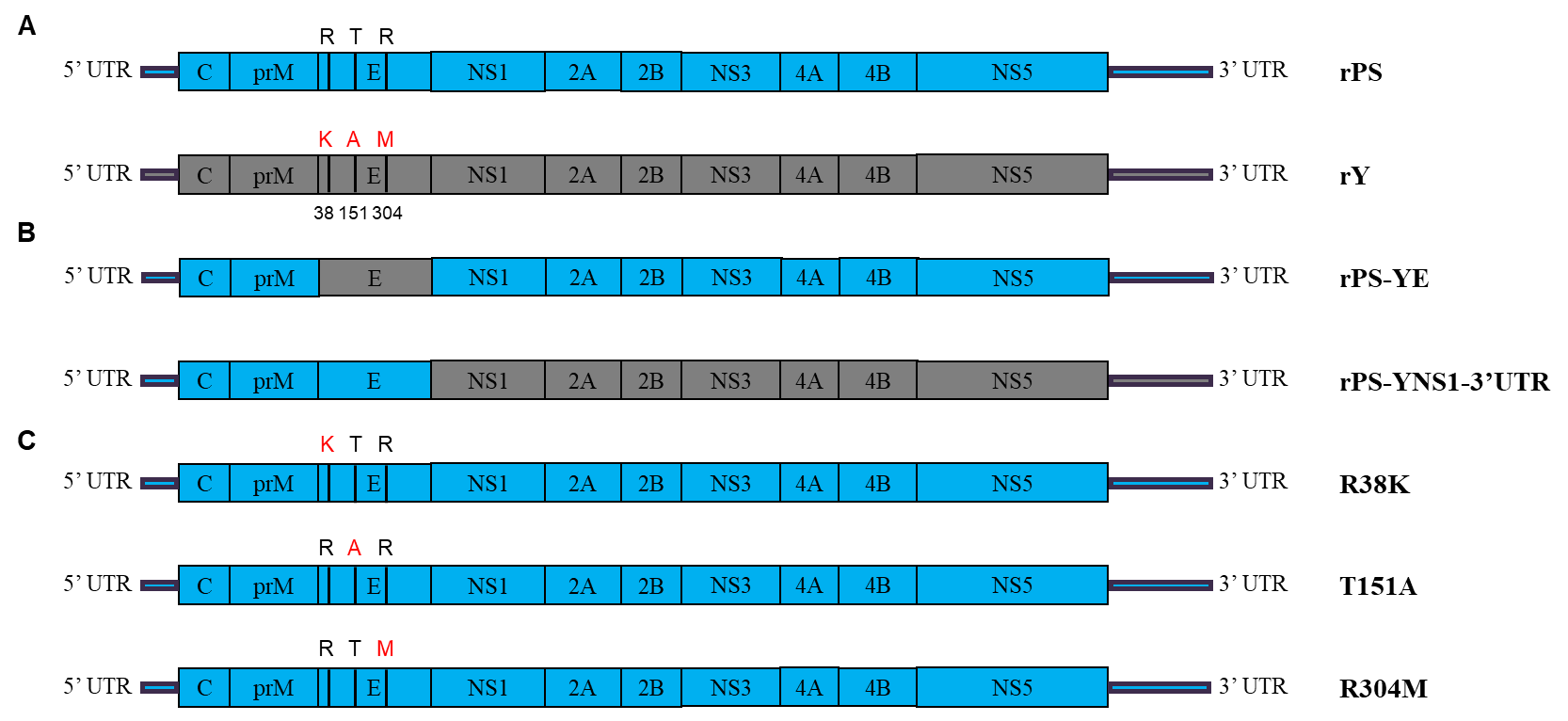


**Supplementary Figure 1.** Diagram of genomes of parental (**A**), chimeric (**B**), and mutant (**C**) viruses. The genes of rPS and rY are indicated with light blue and gray, respectively. Amino acids in the E protein that differed between rPS and rY are indicated with black (rPS) and red (rY) letters above the gene boxes, and their positions in the E protein are shown under the box.


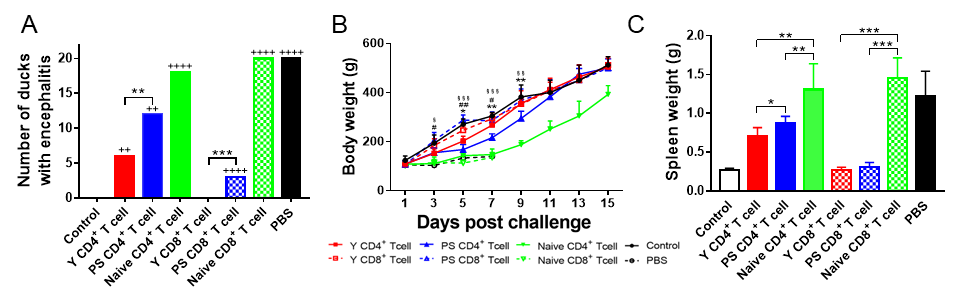


**Supplementary Figure 2.** CD4^+^ and CD8^+^ T cells protect against signs of encephalitis, weight loss, and gross lesions. The adoptive cell transfer protocol was shown in FIGURE 6. **(A)** Number of recipient ducklings exhibiting signs of encephalitis. ++, mild; ++++, marked. **, *P*<0.01; ***, *P*<0.001. **(B)** Measurement of body weight of recipient ducklings. Three individuals in each group were weighed at each time point pi. Data are presented as mean ±SD. Asterisks indicate significant differences between groups received Y- and PS-specific CD4^+^ T cells (*, *P*<0.05; **, *P*<0.01; ***, *P*<0.001). The # signs indicate significant differences between the group received Y-specific CD4^+^ T cells and the control (#, *P*<0.05; ###, *P*<0.001). The § signs indicate significant differences between the group received PS-specific CD4^+^ T cells and the control (§, *P*<0.05; §§, *P*<0.01; §§, *P*<0.001). **(C)** Measurement of spleen weight of recipient ducklings. Spleens of three individuals in each group were weighed at 7 days pi. Data are presented as mean ± SD. *, *P*<0.05; **, *P*<0.01; ***, *P*<0.001.


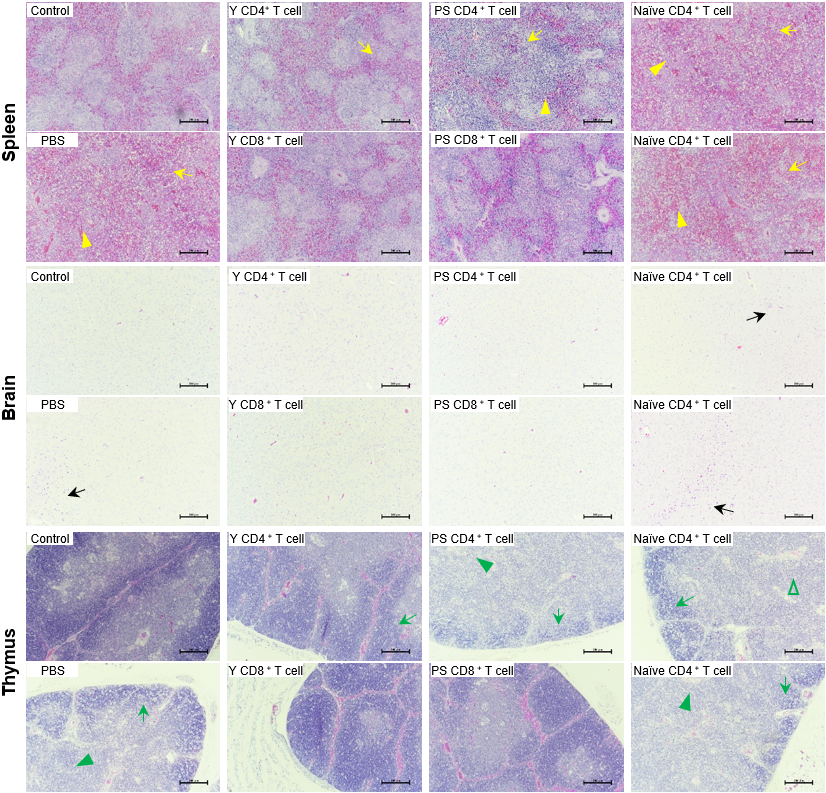


**Supplementary Figure 3.** CD4^+^ and CD8^+^ T cells protect against TMUV-induced tissue injury. The adoptive cell transfer protocol was shown in FIGURE 6. Tissues were collected from recipients at 7 days after challenge. Histopathological changes observed in each tissue were as follows: spleen, necrotic vacuole (yellow arrows) and indistinct interface between red-pulp and white-pulp (yellow triangles); brain, inflammatory cell aggregation (black arrows); and thymus, necrotic vacuole in immature lymphocyte layer (green arrows) and disappearance of thymic corpuscles (green triangles). Bar = 200 μm.
